# Decoding Microbiota in Genitourinary Oncology: Biological Mechanisms and Clinical Implications—A Narrative Review

**DOI:** 10.3390/cancers18030497

**Published:** 2026-02-03

**Authors:** Irene Caramella, Chiara Abeni, Sara Cherri, Chiara Ogliosi, Tonino Morena, Giacomo Galvagni, Fausto Meriggi, Alberto Zaniboni

**Affiliations:** 1 Department of Clinical Oncology, Fondazione Poliambulanza, 25124 Brescia, Italy; irene.caramella@poliambulanza.it (I.C.); fausto.meriggi@poliambulanza.it (F.M.); alberto.zaniboni@poliambulanza.it (A.Z.); 2 Department of Urology, Fondazione Poliambulanza, 25124 Brescia, Italy

**Keywords:** microbiota, genitourinary malignancies, gut–urogenital axis, intratumoral microbiome, immune checkpoint inhibitors, precision oncology

## Abstract

Genitourinary malignancies are characterized by marked heterogeneity in tumor biology, clinical behavior and therapeutic outcomes. Despite significant progress in surgical and systemic treatments, resistance to therapy remains a major challenge, highlighting the need to identify additional host-related determinants of disease progression and treatment response. Within this framework, converging experimental and clinical evidence indicates that host-associated microbial ecosystems may influence key biological processes involved in tumor–host interactions, including immune modulation, metabolic regulation and inflammatory pathways. Altered microbial profiles have been associated with oncogenic signaling, changes in the tumor microenvironment and differences in clinical benefit from systemic therapies, particularly immunotherapeutic approaches. This review brings together preclinical, translational and clinical evidence on the involvement of microbiota in renal, prostate, bladder and testicular cancers, with attention to biological mechanisms and clinically meaningful correlations with disease characteristics. While current data are largely observational, early interventional studies suggest that modulation of microbial ecosystems may influence therapeutic activity in selected clinical settings. Collectively, these findings support microbiota as a relevant component of genitourinary cancer biology with potential implications for precision medicine approaches.

## Simple Summary

Genitourinary cancers are common malignancies that often display substantial variability in their clinical course and response to treatment. In recent years, increasing attention has focused on human microbiota as a biological factor capable of influencing inflammatory, immune and metabolic processes. Growing evidence suggests that alterations in microbial communities may contribute to cancer development and modulate therapeutic outcomes. This review provides an overview of current research on the involvement of microbiota in renal, prostate, bladder and testicular cancers. It summarizes key biological mechanisms and clinically relevant observations that have contributed to the rising interest in this field.

## 1. Introduction

Genitourinary malignancies, including prostate, bladder, renal and testicular cancers, represent a major global health concern, accounting for more than two million new cases and nearly 800,000 deaths per year [1]. Despite significant advances in surgical and systemic approaches, disease recurrence and therapeutic resistance remain current challenges. As a result, research efforts are expanding beyond pharmacological interventions to focus on biological factors that modulate tumor evolution and influence heterogeneity in clinical outcomes [2]. Over the past decade, the human microbiome—composed of bacteria, fungi, viruses and archaea—has emerged as a key regulator of host physiology and pathology [3]. Microbial communities colonize a wide range of epithelial and mucosal surfaces, where they engage in dynamic interactions with immune, endocrine and metabolic pathways [4]. Perturbations in these interactions may contribute to carcinogenesis through multiple mechanisms, including disruption of the balance between proliferation and apoptosis, reprograming of immune responses and modulating the metabolism of nutrients, drugs and host-derived factors [5]. Through these pathways, the microbiota can promote tumor development by sustaining chronic inflammation, generating oncogenic metabolites, interfering with DNA repair processes and modulating systemic immunity [5,6]. Beyond its involvement in cancer initiation and progression, microbiota may also exert antitumor effect and influence treatment outcomes, particularly in the context of immunotherapy [7,8]. Microbial products and metabolites have been shown to modulate apoptosis, antigen presentation and T-cell priming within the tumor microenvironment (TME) [9]. Both preclinical and clinical evidence indicates that baseline microbial composition as well as dynamic changes during treatment can affect responses to immune checkpoint inhibitors (ICIs) [10,11]. Taken together, these observations support microbiota as a key determinant of cancer development, progression and therapeutic modulation. Understanding how microbial communities interact with tumor cells across different anatomical niches may provide new opportunities for biomarker discovery, risk stratification and microbiota-guided therapeutic strategies. This review aims to explore the complex interactions between microbiota and genitourinary cancers and the role of microbial dysbiosis in oncogenesis, tumor progression and clinical outcomes.

## 2. Material and Methods

This manuscript is a narrative review based on published preclinical, translational and clinical studies investigating the role of microbiota in genitourinary malignancies. The relevant literature was identified through searches on PubMed, using combinations of the following keywords: renal cell carcinoma, bladder cancer, prostate cancer, testicular cancer, gut microbiome, urinary microbiome, and intratumoral microbiome. Priority was given to peer-reviewed articles published within the last 10 years, with the inclusion of earlier landmark studies if necessary to provide historical context. Studies were selected based on their clinical or biological relevance to tumor development, progression, and treatment response. Non-English articles, isolated case reports and studies not directly related to genitourinary oncology were excluded.

Three major microbial compartments relevant to genitourinary oncology were considered: 1. the gut microbiota, referring to intestinal luminal and mucosa-associated microbial communities; 2. the urinary microbiome (urobiome), representing low-biomass microbial ecosystems detectable in urine and the lower urinary tract; 3. the intratumoral microbiota, defined as microbial DNA or viable microorganisms identified within tumor tissue itself. Clear compartmentalization is essential, as each niche differs in microbial biomass, sampling methodology and biological interpretation.

## 3. Renal Cancer

Renal cell carcinoma (RCC) is one of the most common malignancies and represents a major cause of cancer-related mortality worldwide. Its incidence continues to rise globally, with about 400,000 new cases and 180,000 deaths per year [1]. Characterized by remarkable heterogeneity in histology, molecular drivers and immune landscape, RCC has always represented a major challenge due to its variability in clinical behavior and therapeutic response [12]. Despite significant advances achieved with tyrosine kinase inhibitors (TKIs) targeting VEGF or mTOR pathways and, more recently, by ICIs, a large proportion of patients still experience treatment resistance or only transient benefit [13]. This has prompted the search for novel host-related factors, beyond genetic mechanisms, that influence cancer development, progression, and treatment response—among which the microbiota has emerged as a potential key player.

### 3.1. Gut Microbiota and RCC

**Figure 1:**
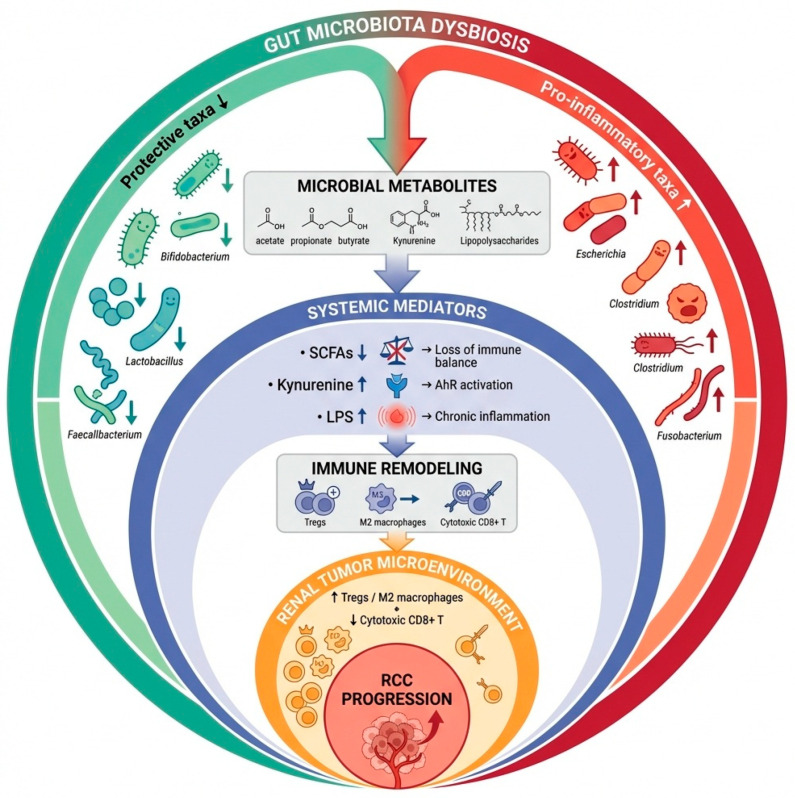
Mechanisms linking gut microbiota dysbiosis and RCC progression.

Growing evidence shows that microorganisms may influence renal physiology through the production of mediators that regulate homeostasis, inflammation and angiogenesis, creating a functional gut–kidney axis [14]. Thus, disruptions in microbial composition can alter biological pathways, contributing to renal carcinogenesis [14]. Several studies have identified distinct microbial signatures associated with RCC risk or progression. In particular, depletion of short-chain fatty acids (SCFAs)-producing taxa, such as members of the Ruminococcaceae, Lachnospiraceae and other butyrate-generating genera, appears to characterize the gut microbiota of RCC patients and is biologically relevant given the immunoregulatory functions of SCFAs [15]. These metabolites, including butyrate and propionate, exert anti-inflammatory effects by enhancing cytotoxic T-cell activity, promoting regulatory T-cell homeostasis and suppressing protumor inflammation through histone deacetylase inhibition and activation of G-protein-coupled receptors such as GPR43 [16]. Their reduction therefore contributes to a tumor-permissive microenvironment marked by chronic inflammation and impaired immune surveillance. Conversely, enrichment of pro-inflammatory and potentially pathogenic taxa, particularly Bacteroides, Escherichia, Fusobacterium, and other members of the Proteobacteria phylum, has been associated with increased susceptibility to RCC and with more aggressive disease patterns [15]. These bacteria can amplify inflammatory signaling, disrupt epithelial integrity and modulate immune checkpoints within the TME, thereby facilitating tumor progression [15]. Microbial metabolites arising from amino acid metabolism further reinforce this imbalance. In particular, tryptophan catabolism along the kynurenine–aryl hydrocarbon receptor (AhR) pathway promotes a markedly pro-inflammatory and pro-tumorigenic state. Accumulation of kynurenine in RCC activates AhR, driving epithelial–mesenchymal transition, enhancing migration and invasion, and reshaping the tumor microenvironment toward immune suppression and chronic inflammation (Figure 1) [17].

### 3.2. Urinary Tract Microbiota and RCC

Microorganisms residing in the urinary tract may also contribute to tumor development. In a small exploratory study of 12 patients with RCC, the urinary microbiome was found to differ significantly from that of healthy controls, with enrichment of Corynebacterium granulosum, Propionibacterium lacydonensis and Thermus arenae [18]. Additionally, recurrent urinary tract infections (UTIs) and kidney stones—conditions frequently associated with microbial imbalance, especially with implication of uropathogens like Escherichia coli and Klebsiella spp.—have been consistently correlated with increased incidence of RCC [19,20,21,22]. Individuals with a history of kidney or bladder infections show nearly a twofold higher risk of RCC (OR = 1.9), and the combination of UTIs with smoking further amplifies this risk (OR = 9.7) [19]. Although direct causal evidence remains limited, these observations suggest that urinary dysbiosis may influence renal carcinogenesis through mechanisms involving chronic inflammation, immune modulation or metabolic reprograming within the renal microenvironment [23].

### 3.3. Intratumoral Microbiota and RCC

The emerging characterization of intratumoral microbiota further adds complexity to RCC biology. Ribosomal RNA sequencing of renal tumors and matched normal tissues consistently reveals a distinct tumor-associated microbiome enriched in genera such as Cutibacterium, Sphingomonas, Roseomonas, Escherichia/Shigella and Fusobacterium, which have been repeatedly identified across independent RCC cohorts [24,25,26,27]. An inverse correlation between declining microbial richness and increasing tumor histological grade further supports the existence of a selective ecological pressure during tumor evolution [24]. Although the functional contribution of these tumor-resident microbes remains only partially understood, accumulating evidence suggests that they may modulate immune checkpoints by altering antigen presentation and by shaping cytokine and chemokine gradients within the tumor microenvironment [15]. In addition, microbial-derived products can act as immune-stimulatory or tolerogenic signals, influencing key immunological processes such as macrophage polarization, dendritic cell maturation, and T-cell recruitment, thereby contributing to immune evasion or immune activation depending on the microbial context [25,26].

### 3.4. Therapeutic Implications and Ongoing Studies

The clinical relevance of microbiota extends to therapeutic responsiveness, particularly in the context of ICIs. Several studies have shown that exposure to antibiotics shortly before or after the initiation of immunotherapy disrupts microbial equilibrium and is associated with reduced progression-free survival (PFS) and overall survival (OS) in metastatic RCC [28,29,30]. Similar detrimental effects have been reported for proton pump inhibitors and corticosteroids, which are known to impair microbiome diversity and immune activation [31,32]. Preclinical xenograft models further indicate that modulation of the gut microbiota can enhance the efficacy of ICIs by promoting antitumor T-cell activation and reprograming the tumor microenvironment [33]. Of note, enrichment of taxa such as Akkermansia muciniphila, along with other commensals identified in broader solid-tumor cohorts (i.e., Bacteroides salyersiae and Eubacterium siraeum), has been associated with improved responses to anti–PD-1 therapy and more favorable immune profiles, patterns that have also been increasingly reported in RCC populations [34,35]. Conversely, dysbiosis characterized by pathogenic species—such as Clostridium hathewayi—correlates with poorer outcomes and diminished ICIs efficacy [36,37]. These observations have stimulated interest in microbiota-targeted therapeutic strategies. In a randomized phase I study, the addition of the butyrate-producing probiotic Clostridium butyricum (CBM588) to nivolumab plus ipilimumab significantly prolonged PFS compared with immunotherapy alone, while restoring microbial diversity and expanding beneficial taxa such as Bifidobacterium and Akkermansia [38]. Similarly, fecal microbiota transplantation (FMT) from ICI responders has demonstrated the ability to re-sensitize refractory or antibiotic-exposed RCC patients to immunotherapy [39]. Several ongoing clinical trials directly evaluate microbiota modulation in RCC. The TACITO trial (NCT04758507) is a randomized phase 1/2 study testing FMT combined with pembrolizumab plus axitinib, with preliminary findings indicating improved response rates and PFS in the FMT arm. The MITRIC trial (NCT05286294) investigates FMT from good responders to overcome acquired resistance to ICIs in refractory RCC, while the BIOFRONT trial (SWOG) assesses CBM588 in combination with frontline nivolumab–ipilimumab in a phase III multicenter setting. Together, these studies reflect a rapidly expanding therapeutic landscape aimed at leveraging the microbiome to improve treatment outcomes in RCC (Table 1). Finally, recent evidence indicates that the gut microbial milieu may also influence responses and toxicities associated with tyrosine kinase inhibitors (TKIs). In patients treated with sunitinib or pazopanib, enrichment of Barnesiella and Akkermansia has been linked to favorable outcomes, whereas dysbiosis has been associated with gastrointestinal toxicity and impaired therapeutic benefit [37,40].

## 4. Prostate Cancer

Prostate cancer (PC) is one of the most frequently diagnosed malignancies among men and remains a leading cause of cancer-related mortality worldwide [1]. Traditionally viewed as a hormonally driven disease, PC is now recognized as a multifactorial malignancy in which environmental, metabolic and immune determinants interact with genetic and epigenetic alterations [41]. Within this framework, the microbiota has been increasingly investigated in relation to its potential association with prostatic homeostasis and tumor biology [42,43].

### 4.1. Gut Microbiota and PC

Growing attention has been directed to the concept of a gut–prostate axis, given the profound impact of intestinal microbes on host metabolism, systemic inflammation and endocrine function [43,44]. Dysbiosis is associated with chronic low-grade inflammation, alterations in lipid and amino acid metabolism and disruption of androgen and estrogen signaling, all of which contribute to a permissive environment for carcinogenesis and disease progression [45]. The gut microbiota also contributes to the synthesis and availability of B-group vitamins, including folate, biotin and riboflavin, which are essential cofactors for one-carbon metabolism, DNA methylation and genomic stability [45]. Studies in PC patients have shown enrichment of folate- and arginine-related pathways in fecal microbiomes compared with controls, suggesting a potential link between microbial vitamin metabolism and epigenetic deregulation in prostate carcinogenesis [41,45,46]. Metagenomic and 16S rRNA sequencing analyses in men with PC versus benign prostatic hyperplasia (BPH) or healthy controls have reported alterations in gut microbial composition, although specific signatures vary across cohorts and methodologies [43]. Several studies describe increased abundance of Bacteroides and Escherichia, together with a reduction in SCFA-producing taxa such as Faecalibacterium prausnitzii and Eubacterium rectale, consistent with a shift from anti-inflammatory toward pro-inflammatory configurations in subsets of patients, as disease risk and aggressiveness increase [45,47]. SCFAs such as butyrate and propionate—produced by these commensals—are key immunomodulatory metabolites; their depletion may weaken immune competence, including cytotoxic T-cell functions, through mechanisms that include histone deacetylase inhibition and broader effects on systemic immunity [48,49]. In parallel, certain Bacteroides species and other gut bacteria express β-glucuronidases capable of deconjugating estrogen metabolites, thereby modulating enterohepatic estrogen recirculation; this process may indirectly influence systemic sex hormone balance and proliferative signaling in hormone-responsive tissues, including the prostate [50]. Diet-induced microbial changes further contribute to this interplay, as high-fat, low-fiber or Western dietary patterns have been associated with endotoxin translocation, oxidative stress and NF-κB activation, linking dietary habits, microbiota remodeling and prostate inflammation and cancer risk [44,51].

### 4.2. Urinary Tract Microbiota and PC

Beyond the intestinal compartment, disease-associated microbial signatures have also been detected in the urinary tract. Next-generation sequencing (NGS) studies reveal distinct urinary microbiome profiles in men with PC compared with healthy or BPH controls, with a relative enrichment of potential uropathogens and pro-inflammatory taxa [52,53]. Reported findings include increased abundance of species such as Streptococcus anginosus, Anaerococcus lactolyticus, Actinobaculum schaalii and Propionimicrobium lymphophilum, whereas commensal genera including Lactobacillus, Faecalibacterium and Corynebacterium tend to be reduced, although specific signatures vary across cohorts and methodologies [18,52,53]. These organisms may contribute to mucosal homeostasis and local immune defense, and their depletion favors a pro-inflammatory microenvironment in the lower urinary tract.

### 4.3. Intratumoral Microbiota and PC

At a local level, microbial DNA and components have been detected within prostate tissue itself, challenging the traditional notion of the prostate as a sterile organ [54]. Multiple sequencing studies report the presence of bacterial genera such as Cutibacterium (Propionibacterium) acnes, Staphylococcus and Pseudomonas within PC specimens, although low biomass and contamination control remain critical methodological issues in interpreting tissue microbiome data [52,54,55,56]. In particular, C. acnes has been implicated in chronic prostatic inflammation and prostatitis-related pathways, potentially through mechanisms involving induction of oxidative stress, recruitment of regulatory T cells and increased production of anti-inflammatory cytokines such as interleukin (IL)-10 and transforming growth factor-β (TGF-β) [55,57]. Mechanistically, microbial products such as lipopolysaccharides (LPS) and other pathogen-associated molecular patterns (PAMPs) can activate Toll-like receptor (TLR) signaling in epithelial and immune cells, inducing NF-κB activation and pro-inflammatory cytokine production (e.g., IL-6, IL-8, and TNF-α), thereby sustaining a tumor-promoting inflammatory milieu [58,59]. Additional evidence suggests that intratumoral microbes can modulate innate and adaptive immune cell phenotypes, potentially contributing to immunosuppressive niches that favor tumor progression [60].

### 4.4. Therapeutic Implications and Ongoing Studies

Beyond tumorigenesis, microbiota has emerged as a determinant of treatment response in PC, particularly in relation to androgen deprivation therapy (ADT) and, to a lesser extent, ICIs [61]. Translational evidence suggests that ADT may influence gut microbiota indirectly through profound alterations in systemic sex hormone levels that contribute to systemic inflammatory and metabolic impairment associated with hormonal treatment [61]. With regard to ICIs, evidence in PC remains limited compared with RCC; however, broader solid-tumor analyses, primarily derived from melanoma and lung cancer cohorts, suggest that reduced microbial diversity and enrichment of pro-inflammatory taxa may associate with impaired ICI efficacy, whereas microbiomes enriched in SCFA-producing or mucin-degrading commensals may correlate with more favorable immune profiles—observations that require validation in PC-specific cohorts [37]. These associations should therefore be considered largely extrapolative in prostate cancer and require prospective validation in PC-specific cohorts. In this context, microbiota-targeted strategies have been proposed as adjuncts to systemic therapy in PC, including dietary modulation, probiotics, prebiotics/postbiotics and FMT [52]. In metastatic castration-resistant prostate cancer (mCRPC), a phase I/II trial (NCT04116775) is evaluating FMT from responders to pembrolizumab in men who have not responded to pembrolizumab plus enzalutamide, with the aim of restoring a favorable microbial configuration and re-sensitizing tumors to ICI therapy. Another ongoing study (NCT06616597) targets gut bacterial androgen-related mechanisms to reverse resistance to abiraterone/prednisone in mCRPC (testing dexamethasone ± metronidazole with abiraterone). In addition, the AkkPRO study (NCT06242509) investigates Akkermansia muciniphila in advanced PC, assessing safety and microbiome effects in the context of abiraterone-associated microbiome modulation (Table 1).

## 5. Bladder Cancer

Bladder cancer (BC), predominantly urothelial carcinoma, represents one of the most common malignancies of the genitourinary tract, with nearly 600,000 new cases and more than 200,000 deaths reported annually worldwide [1]. BC is characterized by marked clinical and biological heterogeneity, ranging from non-muscle-invasive tumors with high recurrence rates to aggressive muscle-invasive and metastatic disease. Despite well-established environmental risk factors, chronic inflammation and immune dysregulation are increasingly recognized as central contributors to urothelial carcinogenesis and disease progression [62]. In this context, emerging evidence implicates microbiota as a relevant modulator of BC biology and therapeutic response.

### 5.1. Gut Microbiota and BC

Growing evidence supports the existence of a gut–bladder axis, whereby intestinal microbiota influences systemic inflammation, immune tone and metabolic homeostasis, indirectly modulating urothelial carcinogenesis [62]. Dysbiosis may impair epithelial barrier integrity and increase gut permeability, facilitating the translocation of microbial-derived molecules, such as LPS, into systemic circulation, where they can affect distant organs, including the bladder [63]. These signals may contribute to chronic low-grade inflammation and immune dysregulation, creating a permissive environment for tumor initiation and progression. Diet-induced alterations in gut microbial composition further shape this axis [64]. Western dietary patterns have been associated with reduced production of SCFAs, altered bile acid metabolism and increased systemic inflammatory signaling, whereas fiber-rich diets promote SCFA-producing commensals with anti-inflammatory and immunoregulatory properties [64]. In the context of BC, preclinical models based on N-butyl-N-(4-hydroxybutyl)nitrosamine (BBN) underscore the central role of inflammatory signaling in tumor development, while microbial perturbations have been proposed as modulators of host responses to carcinogenic exposure rather than direct determinants of tumor initiation [65].

### 5.2. Urinary Tract Microbiota and BC

Beyond systemic influences, the urinary tract itself harbors a distinct microbial ecosystem. In contrast to the historical assumption of urine sterility, culture-independent studies have demonstrated the presence of a structured urinary microbiota in healthy individuals, with disease-specific alterations in bladder cancer [66,67,68,69]. Multiple independent cohorts have reported significant differences in urinary microbial diversity and composition between BC patients and controls, as well as across disease stages [70]. BC-associated urinary dysbiosis is frequently characterized by enrichment of potentially pro-inflammatory or pathogenic taxa, including Acinetobacter, Streptococcus, Enterococcus, Klebsiella and Fusobacterium, alongside depletion of commensal genera such as Lactobacillus and Corynebacterium [66,69]. These microbial shifts are thought to promote a pro-inflammatory luminal milieu, potentially sustaining chronic urothelial irritation and immune activation. Independent studies have demonstrated associations between altered urinary microbiota profiles, tumor presence and disease stage, supporting a link between urinary dysbiosis and urothelial carcinogenesis [67,70]. Urinary microbiota composition has also been linked to clinical outcomes. In patients with non-muscle-invasive bladder cancer (NMIBC), specific urinary microbial signatures have been associated with disease recurrence following transurethral resection, suggesting a potential role for the urinary microbiome as a biomarker of recurrence risk and therapeutic response [68]. While these findings are derived from observational studies, they support the hypothesis that luminal microbial communities may influence tumor persistence and relapse.

### 5.3. Intratumoral Microbiota and BC

Microbial DNA and bacterial components have been detected within bladder tumor tissue [71]. Sequencing analyses of bladder cancer specimens have identified intratumoral microbial communities distinct from adjacent nontumoral tissue, with enrichment of genera such as Pseudomonas, Acinetobacter, Sphingomonas and members of the Enterobacteriaceae [72]. Tissue-based studies have shown that intratumoral microbiota correlate with clinicopathological features of BC, supporting biological relevance beyond passive contamination [71]. Mechanistically, tumor-associated microbes may influence bladder cancer biology through activation of pattern recognition receptors, including Toll-like receptors, leading to NF-κB signaling, cytokine release and immune cell recruitment within the tumor microenvironment [66,72]. More recent evidence suggests that intratumoral bacteria may also contribute to epigenetic reprograming in BC, influencing DNA methylation patterns and chromatin accessibility in cancer-related genes and intersecting with established oncogenic pathways [73]. Nonetheless, given the low microbial biomass of bladder tissue, rigorous contamination controls and methodological standardization remain essential, and causal relationships between intratumoral microbes and bladder carcinogenesis have yet to be definitively established.

### 5.4. Therapeutic Implications and Ongoing Studies

The clinical relevance of microbiota in BC is most evident in the context of immunotherapy. Intravesical Bacillus Calmette–Guérin (BCG) remains the standard of care for high-risk NMIBC, and its antitumor activity is intrinsically linked to immune activation triggered by microbial exposure. Accumulating evidence indicates that host microbial composition, both at the gut and urinary levels, may modulate local and systemic immune responses, thereby influencing the efficacy of BCG therapy and clinical outcomes [74]. Preclinical and translational studies suggest that specific commensal bacteria can enhance BCG-induced antitumor immunity by promoting Th1-polarized responses, increasing cytotoxic T-cell infiltration and strengthening innate immune activation [74,75]. These observations have provided a strong biological rationale for investigating microbiota–BCG interactions in clinical settings. In this context, prospective bladder-specific studies are beginning to emerge (Table 1). Notably, an ongoing observational clinical trial (NCT06153849) is evaluating the association between urinary microbiome composition and response to intravesical BCG in patients with NMIBC, with the aim of identifying microbial signatures predictive of treatment response and recurrence risk. Beyond microbiome profiling, early interventional strategies aimed at modulating host microbial communities are also being explored. A phase IV interventional study (NCT05220124) is currently investigating probiotic supplementation in combination with ICIs in patients with BC. This trial evaluates the administration of a live combined probiotic formulation alongside standard immunotherapy, with the objective of assessing whether microbiota modulation can enhance treatment efficacy and improve PFS. In line with observations in other genitourinary malignancies, emerging evidence suggests that microbiota composition may influence responsiveness to ICIs. Reduced microbial diversity and enrichment of dysbiotic or pro-inflammatory taxa have been associated with impaired antitumor immunity, whereas microbiomes enriched in immunostimulatory and SCFAs-producing commensals appear to correlate with more favorable immune profiles and improved immunotherapy outcomes [75]. However, microbiota-interventional trials in BC remain limited, and further prospective, adequately powered studies are needed to define causality, identify optimal microbial targets and determine the clinical utility of microbiota-guided therapeutic strategies in this disease.

## 6. Testicular Cancer

Testicular germ cell tumors (TGCTs), although relatively rare, represent the most prevalent solid malignancy among young adult males. Despite the high curability of this disease, with survival rates exceeding 95%, resistance to platinum-based chemotherapy remains a significant clinical challenge and is associated with markedly poorer prognosis [76]. Beyond genetic and developmental determinants, the microbiome has recently emerged as a potential modulator of endocrine and immune pathways relevant to testicular physiology and oncogenesis [77].

### 6.1. Gut Microbiota and TGCT

Growing evidence supports the existence of a bidirectional gut–testis axis, whereby intestinal microbial communities influence systemic endocrine signaling and immune homeostasis, with potential implications for testicular tumor biology [77]. Experimental models demonstrate that alterations in gut microbiota composition can modify circulating androgen levels, impair steroidogenesis and reshape the tolerogenic immune environment of the testis [78,79,80]. Through mechanisms that include increased intestinal permeability and translocation of microbial-derived products such as LPS, dysbiosis may disturb steroidogenic pathways and contribute to an inflammatory or hormonally imbalanced milieu conducive to tumor initiation or progression. Conversely, androgens synthesized in the testis can influence gut microbial composition, suggesting a reciprocal regulatory loop between host endocrine function and microbial ecology [81]. Microbial metabolites further contribute to this crosstalk. SCFAs and indole derivatives derived from gut bacteria have been shown to modulate Leydig cell function, local immune tone and germ cell maturation, linking microbial metabolism to testicular homeostasis [79,80]. In addition, microbial-derived inosine enhances Th1 polarization and interferon-γ (IFN-γ) secretion, providing a mechanistic link between gut microbiota and systemic immunoregulation with potential relevance for antitumor immunity [79].

### 6.2. Urinary Tract Microbiota and TGCT

Alterations in extra-intestinal microbial communities have also been reported in patients with TGCTs. Studies profiling the urinary and seminal microbiota describe reduced microbial diversity and enrichment of genera such as Prevotella, Escherichia and Streptococcus, alongside depletion of commensal taxa including Lactobacillus and Faecalibacterium, which are typically associated with anti-inflammatory and immunoregulatory functions [82]. Although these observations remain largely associative, they suggest that microbial signatures in the urogenital tract may reflect systemic immune–microbial perturbations relevant to testicular pathology. Given the anatomical and immunological separation of the testis, urinary and seminal microbiome alterations are more likely to represent downstream markers of systemic dysbiosis rather than direct drivers of tumorigenesis. Nonetheless, their association with inflammatory and endocrine imbalance supports further investigation into their potential role as non-invasive biomarkers.

### 6.3. Intratumoral Microbiota and TGCT

Whether TGCTs harbor a true intratumoral microbiome remains uncertain. Low-biomass sequencing studies have identified bacterial DNA, including genera such as Pseudomonas and Staphylococcus, in a subset of testicular tumor specimens but not in adjacent benign tissue [83]. However, the biological significance of these findings is unclear and may be confounded by technical limitations inherent to low-biomass microbiome analyses, including contamination and sequencing artifacts. Moreover, the presence of the blood–testis barrier and the immune-privileged status of the testicular microenvironment complicate the interpretation of intratumoral microbial signals. At present, available evidence does not support consistent or functionally relevant intratumoral microbiota in TGCTs, and further studies employing rigorous contamination controls are required. The microbiome may also influence therapeutic sensitivity in TGCTs, particularly in the context of emerging immunotherapeutic strategies for refractory disease. Although ICIs have shown limited efficacy in unselected TGCT populations, they are currently being explored in early-phase clinical trials for platinum-refractory tumors [84]. Preclinical evidence suggests that microbiota may modulate sensitivity to ICIs through endocrine–immune interactions rather than through direct enhancement of antitumor immunity. In murine models, depletion of gut microbiota reduces circulating testosterone levels and downregulates physiological PD-L1 expression in testicular tissue, thereby attenuating the immune-privileged state of the testis and increasing sensitivity to anti-PD-L1 therapy [78]. In this context, enhanced immunotherapy efficacy reflects disruption of intrinsic tolerogenic barriers rather than amplification of effector immune responses. Together with evidence linking microbial metabolites such as inosine to improved Th1-driven immune responsiveness [79], these findings support further investigation into microbiota–immune–endocrine interactions in TGCTs. While microbiota-interventional clinical trials are currently lacking in this disease, these mechanistic insights provide a strong rationale for integrating microbiome profiling into future translational and therapeutic studies.

## 7. Conclusions and Future Perspectives

Across genitourinary malignancies, microbiota has emerged as an integral component of tumor biology, shaping oncogenic signaling, immune surveillance and therapeutic responsiveness. Evidence accumulated over the past decade demonstrates that microbial communities are not passive bystanders but dynamic regulators of epithelial homeostasis, metabolism and host immunity [85]. Through complex interactions with hormonal and metabolic pathways, dysbiosis can foster chronic inflammation, oxidative stress and immune evasion—key hallmarks of tumor progression in genitourinary cancers. The detection of microbial DNA also in urine and tumor tissues has substantially changed our understanding of urogenital physiology, overturning the long-held assumption of urinary tract sterility. Each organ appears to host its own niche of microorganisms, whose disruption may trigger or sustain carcinogenic processes (Table 2).

Particularly compelling is the emerging concept of a gut–urogenital axis, in which microbial metabolites—such as SCFAs, bile acids, and tryptophan derivatives—act as systemic messengers integrating immune, endocrine and metabolic circuits, ultimately affecting tumor development and treatment outcomes [86]. Tumors themselves may also reshape TME, suggesting the reciprocal nature of host–microbe interactions. From a clinical perspective, microbiota composition is increasingly recognized as a predictive biomarker of therapeutic efficacy [87]. Distinct microbial profiles have been linked to responses to ICIs, while antibiotic-induced dysbiosis consistently correlates with poorer outcomes across tumor types [88]. Several studies demonstrate that microbial communities exert a direct influence on the host immune landscape, enhancing antigen presentation and T-cell priming, while modulating cytokine production and strengthening antitumor immunity [89]. Conversely, reduced microbial diversity or expansion of pro-inflammatory and immunosuppressive species can disrupt this equilibrium, contributing to immune exhaustion and therapeutic resistance. The discovery of tumor-resident microbiota adds an additional layer of complexity, suggesting that local microbial ecosystems may influence stromal remodeling, antigenicity and the distribution of immune cell populations within the TME. Despite the growing knowledge about microbiome across genitourinary malignancies, several key limitations must be acknowledged. Discerning causality from correlation remains one of the field’s greatest challenges, as most available human studies are observational, and it is still unclear whether microbial alterations precede tumor initiation or instead reflect downstream metabolic, inflammatory, and immunological consequences of malignancy and its treatments. Methodological issues are particularly critical in urinary and intratumoral microbiome analyses, which represent low-biomass settings highly vulnerable to contamination from reagents, skin flora, and sampling procedures, as well as sequencing and bioinformatic biases. In addition, major confounding factors, including antibiotic exposure, proton pump inhibitors, corticosteroids, diet, comorbidities, prior infections, and systemic therapies, can profoundly reshape microbial communities and may partly account for inter-study heterogeneity [90]. To facilitate cross-study comparison and improve interpretability, key clinical and methodological characteristics of representative gut, urinary, and intratumoral microbiome cohorts discussed in this review are summarized in Supplementary Tables S1–S3. Addressing these challenges will require rigorous negative controls, standardized sampling and analytical workflows, longitudinal designs, and validation across independent cohorts to ensure robustness and clinical interpretability of microbiome-related findings.

Preclinical insights underscore that the microbiota functions not only as a biomarker of treatment response but also as a determinant of immune competence and a promising target for therapeutic modulation. Microbial composition contributes to patient-to-patient heterogeneity and may ultimately inform new strategies for precision immuno-oncology. However, current clinical trials are mainly explorative, and further research should be implemented in order to produce practice changing evidence and guidelines. Looking ahead, microbiota modulation offers a promising frontier for precision oncology. Interventions such as dietary modulation, targeted probiotics, postbiotics and FMT may help restore eubiosis, enhance immune competence and mitigate treatment-related toxicities. Integrating multi-omic datasets will be essential to unravel causal mechanisms and identify actionable microbial signatures. The development of “microbiota-informed” therapeutic algorithms may ultimately enable personalized immuno-oncology strategies that optimize therapeutic benefit and long-term outcomes.

In conclusion, the interplay between microbial ecosystems and the urogenital tract represents a rapidly evolving frontier in oncology. By bridging microbial ecology with translational oncology, future research has the potential to redefine how we assess risk, predict treatment response and design novel microbiota interventions, ultimately transforming the clinical management of genitourinary malignancies.

## Figures and Tables

**Table 1:** Ongoing studies in genitourinary malignancies.

Cancer Type	Trial (ID)	Phase	Microbiota Intervention	Treatment Regimen	Primary Endpoint
**RCC**	TACITO (NCT04758507)	I/II	FMT	Pembrolizumab + axitinib	1-year PFS
**RCC**	MITRIC (NCT05286294)	II	FMT	PD after ICIs	Safety and ORR
**RCC**	BIOFRONT (SWOG)	III	*Clostridium butyricum* (CBM588)	Nivolumab + ipilimumab	Effect on relative abundance of gut microbial populations
**mCRPC**	NCT04116775	I/II	FMT	Pembrolizumab (after PD on enzalutamide)	PSA50 (≥ 50% decline in PSA)
**mCRPC**	NCT06616597	II	Antibiotic-based modulation of gut bacterial androgen metabolism (metronidazole)	Abiraterone + dexamethasone	PSA30 (≥ 30% decline in PSA)
**NMIBC**	NCT06153849	Observational	Urinary microbiome profiling	BCG	Association between urinary microbiome composition and response/recurrence after BCG
**MIBC**	NCT05220124	IV	Probiotic supplementation (live combined *Bifidobacterium*, *Lactobacillus*, *Enterococcus*)	ICIs	PFS

RCC—renal cell carcinoma; mCRPC—metastatic castration resistant prostate cancer; NMIBC—non-muscle invasive bladder cancer; MIBC—muscle invasive bladder cancer; FMT—fecal microbiota transplant; PD—progressive disease; ICIs—immune-checkpoint inhibitors; PFS—progression-free survival; ORR—overall response rate; PSA—prostate specific antigen.

**Table 2:** Role of microbiota composition in genitourinary malignancies.

Cancer Type	Tissue/Compartment	Microorganisms spp.	Role in Carcinogenesis	Biological Effect
**RCC**	Gut microbiome	*Bacteroides*, *Escherichia, Fusobacterium* [15]	Pro-carcinogenic	Pro-inflammatory
		*Ruminococcaceae, Lachnospiraceae* [15]	Potentially protective	Anti-inflammatory
		*Akkermansia muciniphila* [34,35,38]	Context-dependent (favors host–tumor interaction)	Anti-inflammatory/immune modulating
	Urinary microbiome	*Escherichia coli, Klebsiella* [19,20,21,22]	Pro-carcinogenic	Pro-inflammatory/immune-modulating
	Tumor tissue	*Cutibacterium*, *Sphingomonas*, *Fusobacterium* [24,25,26,27]	Pro-carcinogenic	Pro-inflammatory
**PC**	Gut microbiome	*Bacteroides*, *Escherichia* [45,47]	Pro-carcinogenic	Pro-inflammatory
		*Faecalibacterium prausnitzii*, *Eubacterium rectale* [45,47]	Potentially protective	Anti-inflammatory
	Urinary microbiome/prostatic fluid	*Anaerococcus lactolyticus*, *Actinobaculum schaalii*, *Streptococcus anginosus, Propionimicrobium lymphophilum* [52,53]	Pro-carcinogenic	Pro-inflammatory
	Tumor tissue	*Cutibacterium (Propionibacterium) acnes, Staphylococcus, Pseudomonas* [52,54,55,56]	Pro-carcinogenic	Pro-inflammatory
**BC**	Urinary microbiome	*Acinetobacter*, *Streptococcus*, *Enterococcus*, *Klebsiella, Fusobacterium* [66,69]	Pro-carcinogenic	Pro-inflammatory
		*Lactobacillus*, *Corynebacterium* [66,69]	Potentially protective	Anti-inflammatory/protective
	Tumor tissue	*Pseudomonas, Acinetobacter, Sphingomonas, Enterobacteriaceae* [72]	Pro-carcinogenic	Pro-inflammatory
**TGCT**	Urinary microbiome/semen	*Prevotella*, *Escherichia*, *Streptococcus* [82]	Pro-carcinogenic	Pro-inflammatory
		*Lactobacillus, Faecalibacterium* [82]	Potentially protective	Anti-inflammatory
	Tumor tissue	*Pseudomonas*, *Staphylococcus* [83]	Pro-carcinogenic	Pro-inflammatory

RCC—renal cell carcinoma; PC—prostate cancer; BC—bladder cancer; TGCT—testicular germ cell tumor.

## Data Availability

No new data were created or analyzed in this study. Data sharing is not applicable to this article.

## Supplementary Materials

The following supporting information can be downloaded at: https://www.mdpi.com/article/10.3390/cancers18030497/s1, Table S1. Key gut microbiome studies in genitourinary cancers. Table S2. Key urinary microbiome studies in genitourinary cancers. Table S3. Key intratumoral microbiome studies in genitourinary cancers.
