# Supplementary material for: Decoding Microbiota in Genitourinary Oncology: Biological Mechanisms and Clinical Implications—A Narrative Review
Source: Cancers (Basel). 2026 Feb 3;18(3):497. doi: 10.3390/cancers18030497 (PMC12897120; doi:10.3390/cancers18030497)
Supplement: Supplementary file 1 [file cancers-18-00497-s001.zip › cancers-4118340-supplementary.pdf]

Study	Cancer	n	Sample	Sequencing method	Clinical context
**Routy et al., 2018 [34]**	(NSCLC) RCC/BC	249	Stool	Shotgun metagenomic sequencing	Advanced cancers on ICIs
**Derosa et al., 2020 [35]**	RCC	69	Stool	Shotgun metagenomic sequencing	Advanced RCC on nivolumab
**Dizman et al., 2022 (CBM588) [38]**	RCC	30	Stool	Shotgun metagenomic sequencing	Nivolumab + Ipilimumab ± probiotic
**Sfanos et al., 2018 [46]**	PC	30	Rectal swab	16S rRNA gene sequencing	ADT
**Liss et al., 2018 [45]**	PC	105	Rectal swab	16S rRNA gene sequencing	Pre-biopsy cohort

Table S1. Key gut microbiome studies in genitourinary cancers

NSCLC – non small cell lung cancer; RCC – renal cell carcinoma; BC – bladder cancer; ICIs - immune-checkpoint inhibitors; PC – prostate cancer; PFS – progression free survival; ADT – androgen deprivation therapy.

Study	Cancer	n	Sample	Sequencing method	Clinical context
**Ahn et al., 2022 [18]**	RCC/BC/PC	85	First-void urine (day of surgery)	16S rRNA (V3–V4)	Prospective surgical cohort
**Shrestha et al., 2018 [52]**	PC	129	VB1 urine (pre-biopsy)	16S rRNA (V6)	Men undergoing prostate biopsy
**Wu et al., 2018 [66]**	BC	49	Mid-stream urine	16S rRNA (V4; MiSeq)	Case–control
**Mansour et al., 2020 [69]**	BC	10	Bladder urine collected during TUR	16S rRNA (V3–V4; MiSeq)	Surgical cohort (paired sampling)

Table S2. Key urinary microbiome studies in genitourinary cancers

RCC – renal cell carcinoma; BC – bladder cancer; PC – prostate cancer.

Study	Cancer	n	Sample	Sequencing method	Clinical context
**Nejman et al., 2020 [83]**	Pan-cancer	1526	Tumor tissue	16S rDNA sequencing + validation (ISH/qPCR)	Atlas study
**Liss et al., 2020 [24]**	RCC	6	Tumor/thrombus	RNA-seq–based microbial signature inference	Nephrectomy specimens
**Cavarretta et al., 2017 [56]**	PC	16	Prostate tissue	Ultradeep 16S rRNA gene sequencing	Prostatectomy specimens

Table S3. Key intratumoral microbiome studies in genitourinary cancers

RCC – renal cell carcinoma; PC – prostate cancer.
